# Supplementary material for: Liquid metals for boosting stability of zeolite catalysts in the conversion of methanol to hydrocarbons
Source: Nat Commun. 2024 Mar 12;15:2228. doi: 10.1038/s41467-024-46232-9 (PMC10933255; doi:10.1038/s41467-024-46232-9)
Supplement: Supplementary file 1 — Supplementary Information [file 41467_2024_46232_MOESM1_ESM.pdf]

Liquid Metals for Boosting Stability of Zeolite Catalysts in the Conversion of Methanol to Hydrocarbons

Yong Zhou,[a,b] Sara O. Santos,[c] Mariya Shamzhy,[d] Maya Marinova,[e] Anne-Marie Blanchenet,[f] Yury Kolyagin,[a] Pardis Simon,[e] Martine Trentesaux,[a] Sharmin Sharna,[g] Ovidiu Ersen,[g] Vladimir L. Zholobenko,[h] Mark Saeys,[c] Andrei Y. Khodakov,[a]* Vitaly V. Ordomsky [a]*

[a] *Univ. Lille, CNRS, Centrale Lille, ENSCL, Univ. Artois, UMR 8181 – UCCS – Unité de Catalyse et Chimie du Solide, F-59000 Lille, France*

[b] *Research Institute of Interdisciplinary Sciences (RISE) and School of Materials Science & Engineering, Dongguan University of Technology, Dongguan 523808, China*

[c] *Laboratory for Chemical Technology (LCT), Department of Materials, Textiles and Chemical Engineering, Ghent University, Technologiepark 125, 9052 Ghent, Belgium;*

[d] *Department of Physical and Macromolecular Chemistry, Faculty of Science, Charles University, Hlavova 2030/8, 12843 Prague, Czech Republic*

[e] *Institut Michel-Eugène Chevreul, 59655 Villeneuve-d'Ascq, France*

[f] *Univ. Lille, CNRS, UMR 8207 - UMET - Unité Matériaux et Transformations, Lille, F-59000, France*

[g] *IPCMS, Université de Strasbourg – CNRS, 67034 Strasbourg, France*

[h] *School of Physical and Chemical Sciences, Keele University, Staffordshire, UK*

Corresponding author: Andrei Khodakov (andrei.khodakov@univ-lille.fr), Vitaly V. Ordomsky (vitaly.ordomsky@univ-lille.fr)

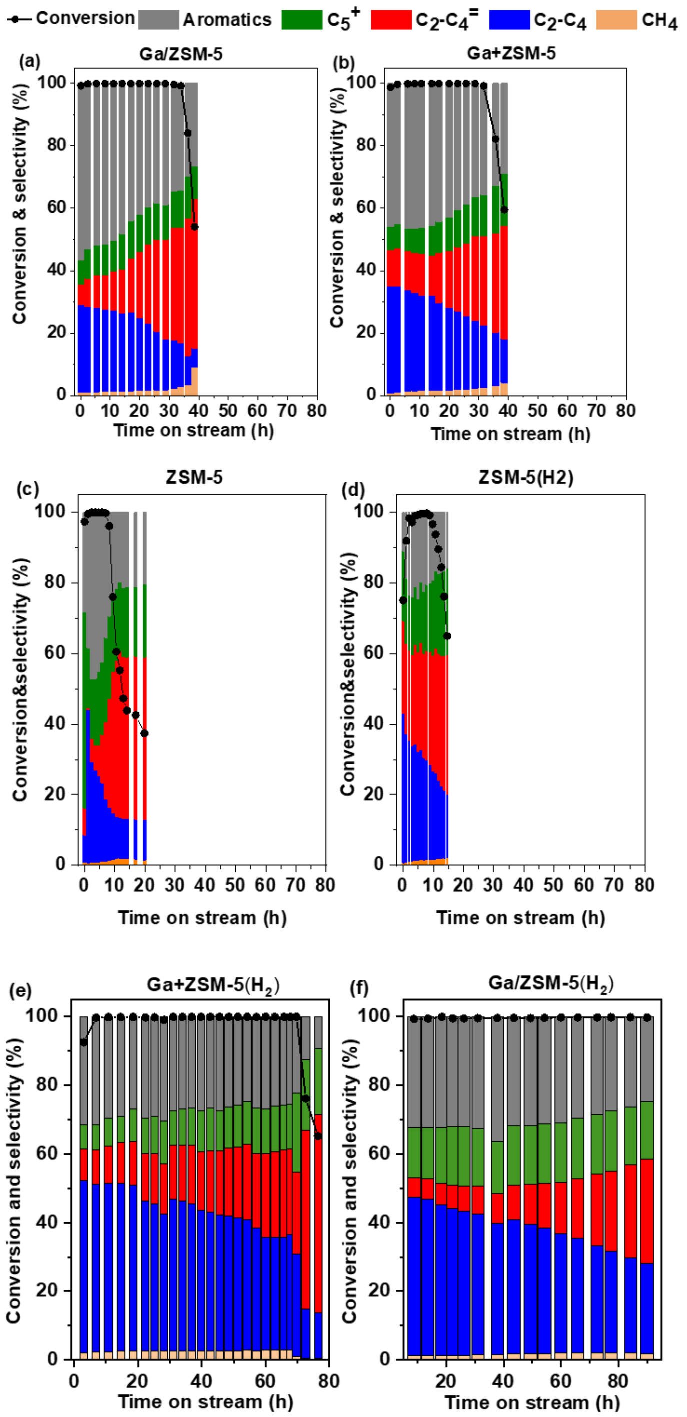

Figure S1. Conversions and selectivities versus time on stream for Ga/ZSM-5 (a, f), Ga+ZSM-5(b, e), H-ZSM-5(c, d) catalysts without hydrogen and in hydrogen flow. Reaction conditions: 400 ℃, catalyst containing 50 mg of ZSM-5, 1.4 g methanol $\cdot g^{-1}_{catalyst\ or\ ZSM\text{-}5}\cdot hour^{-1}$

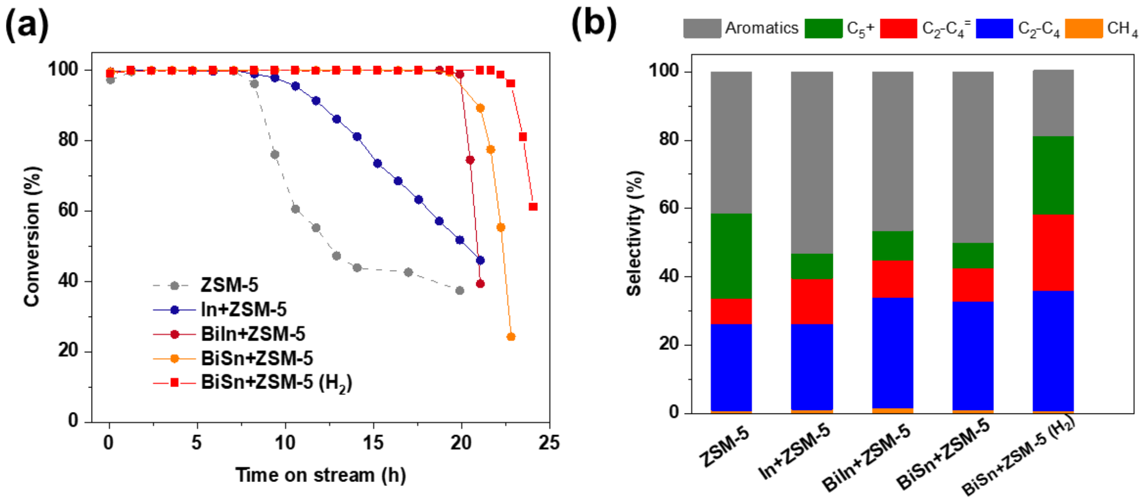

Figure S2. Conversion versus time on stream (a) and products selectivity (b) for ZSM-5 mixing with different liquid metals. Reaction conditions: 400 °C, catalyst containing 50 mg of ZSM-5, 1.4 g methanol $\cdot g^{-1}{}_{ZSM\text{-}5}\cdot hour^{-1}$.

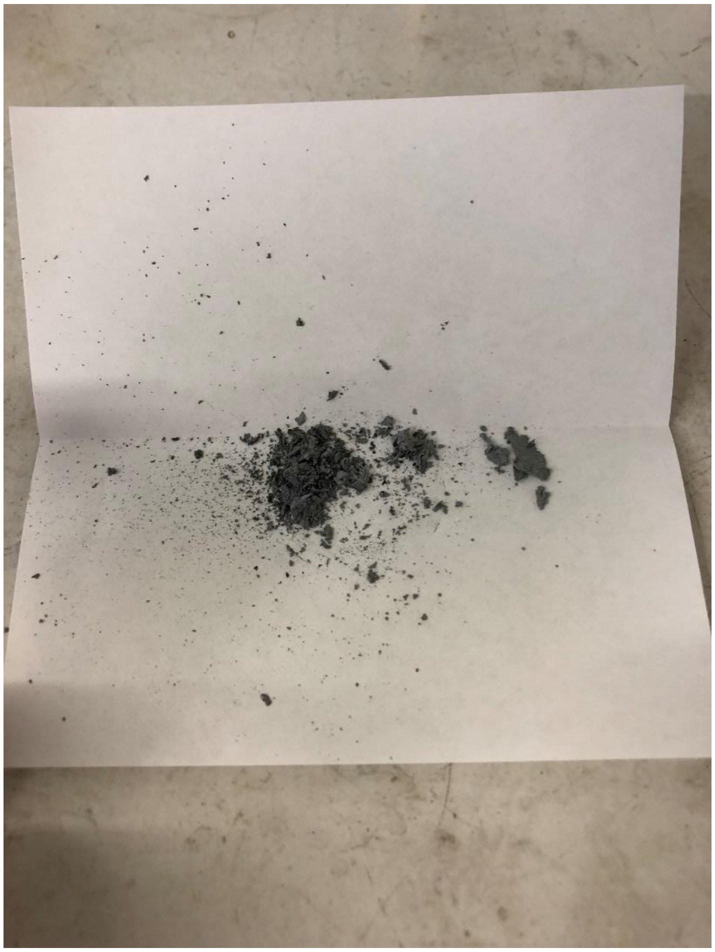

Figure S3. Photo of ZSM-5 treated by Ga for preparation of Ga/ZSM-5 catalyst.

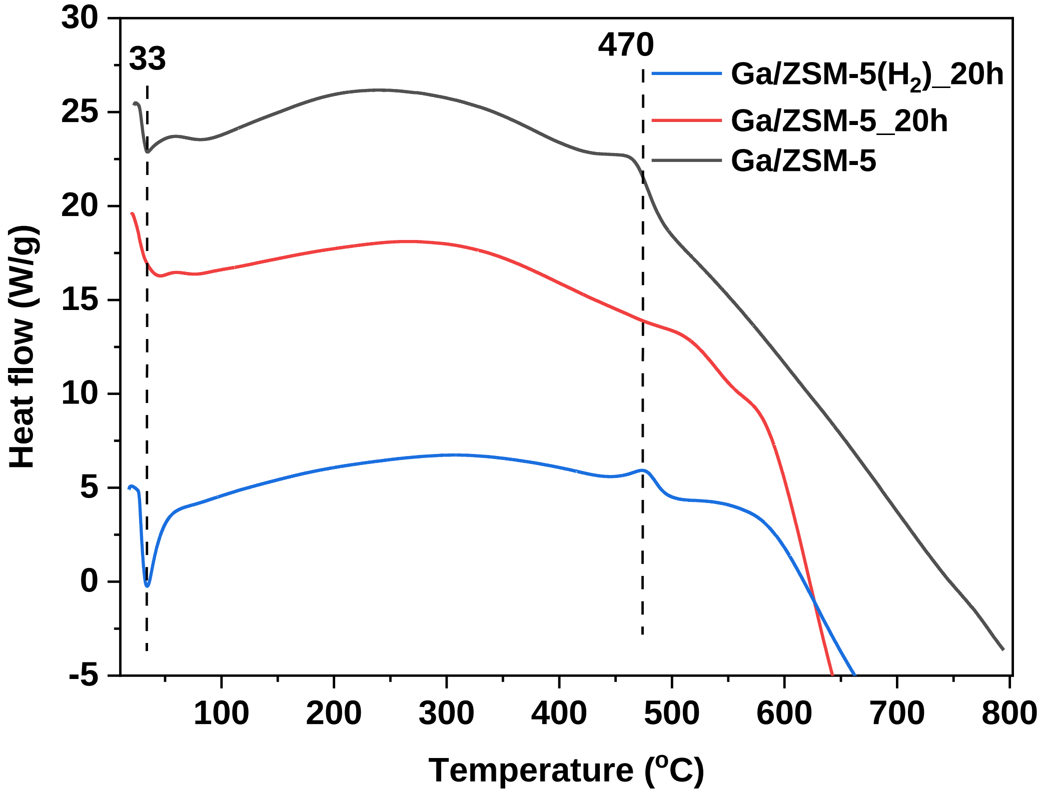

Figure S4. Heat flow during TGA analysis of Ga/ZSM-5 before and after reaction

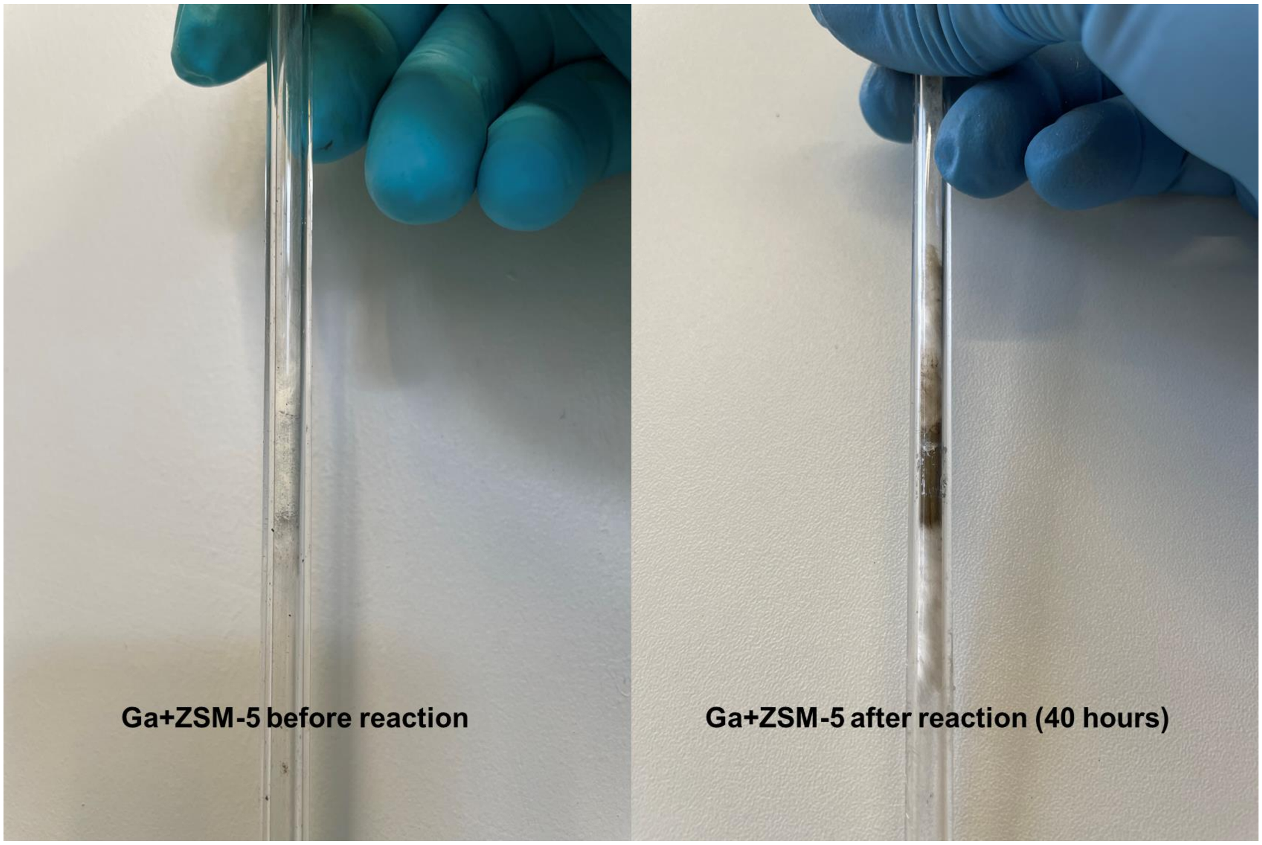

Figure S5. Photo of Ga+ZSM-5 before and after reaction for 40 h.

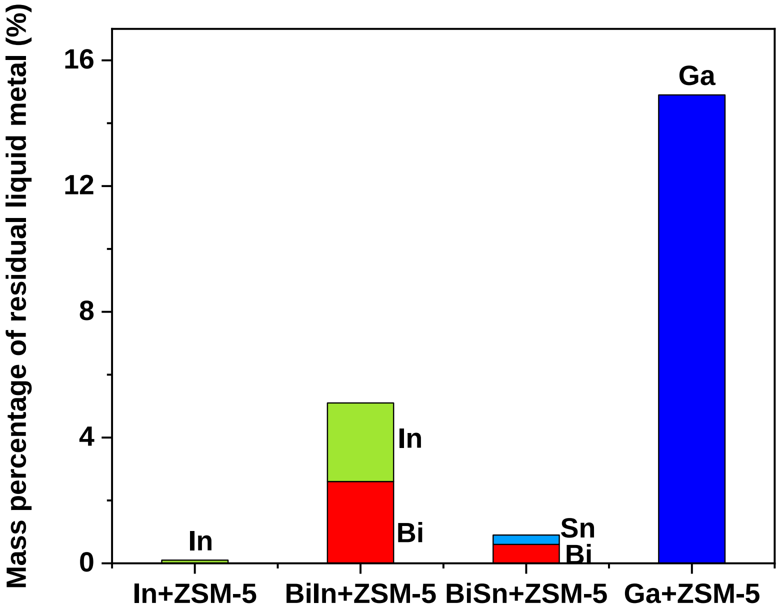

Figure S6. The residual amount of liquid metal on the spent catalysts determined by XRF.

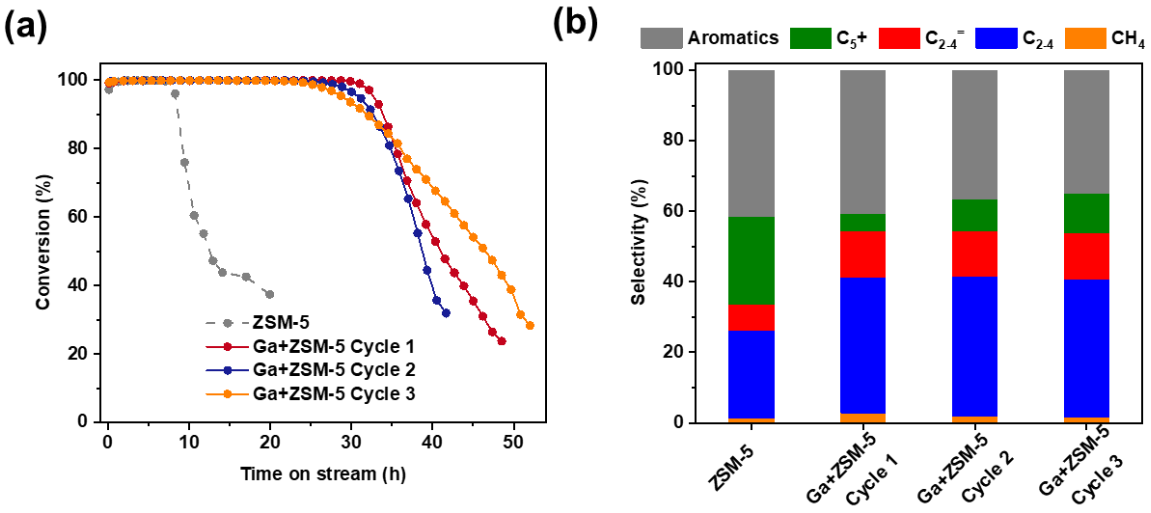

Figure S7. The conversion in time (a) and selectivity (b) over Ga+ZSM-5 for 3 cycles with intermediate calcination at 500 °C for 6h and reduction in hydrogen flow at the same conditions. Reaction conditions: 400 °C, catalyst containing 50 mg of ZSM-5, 1.4 g methanol $\cdot g^{-1}_{ZSM-5} \cdot hour^{-1}$.

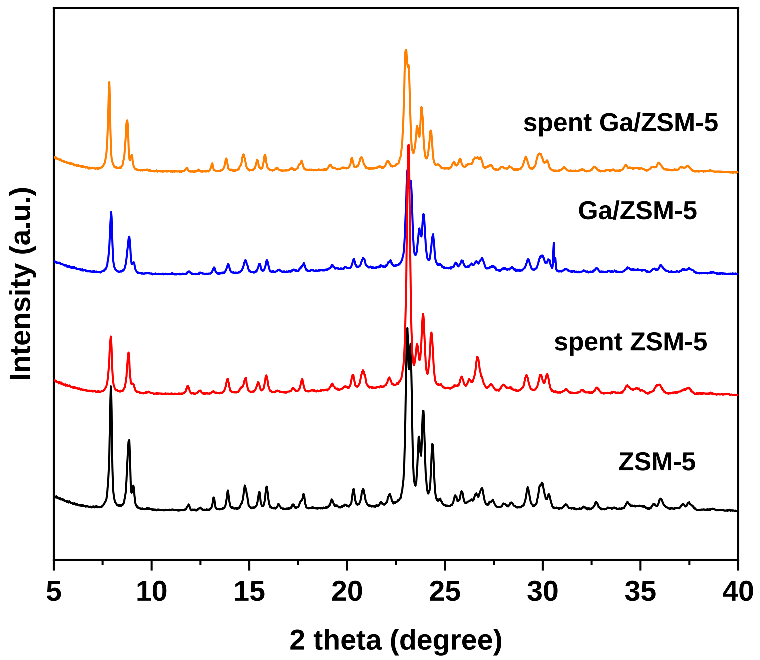

Figure S8. XRD pattens of ZSM-5 and Ga/ZSM-5. Spent samples were obtained after 20 h MTH reaction.

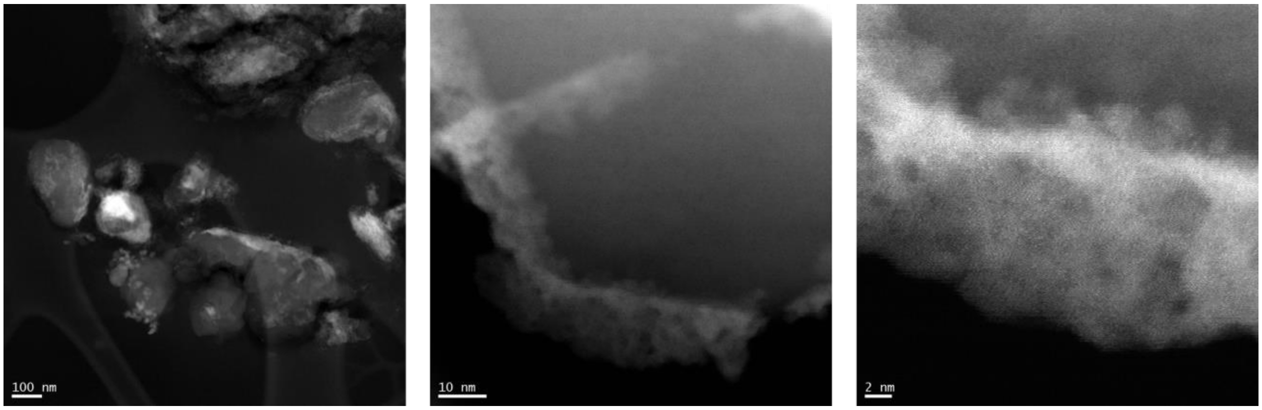

Figure S9. STEM-HAADF images after cutting with cryo-ultramicrotome of Ga/ZSM-5 sample.

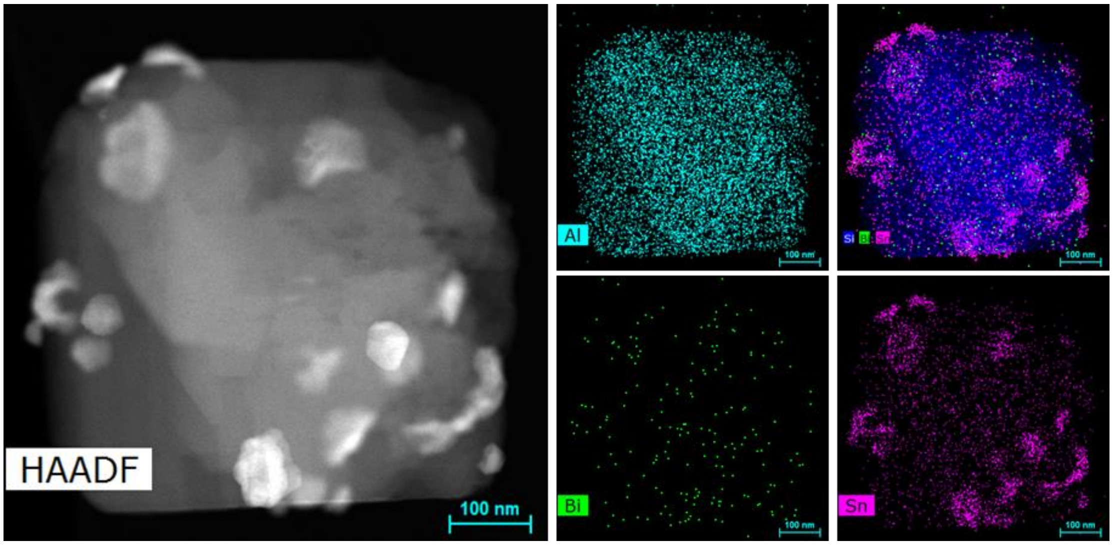

Figure S10. STEM-HAADF and elemental mapping images of BiSn/ZSM-5 catalyst.

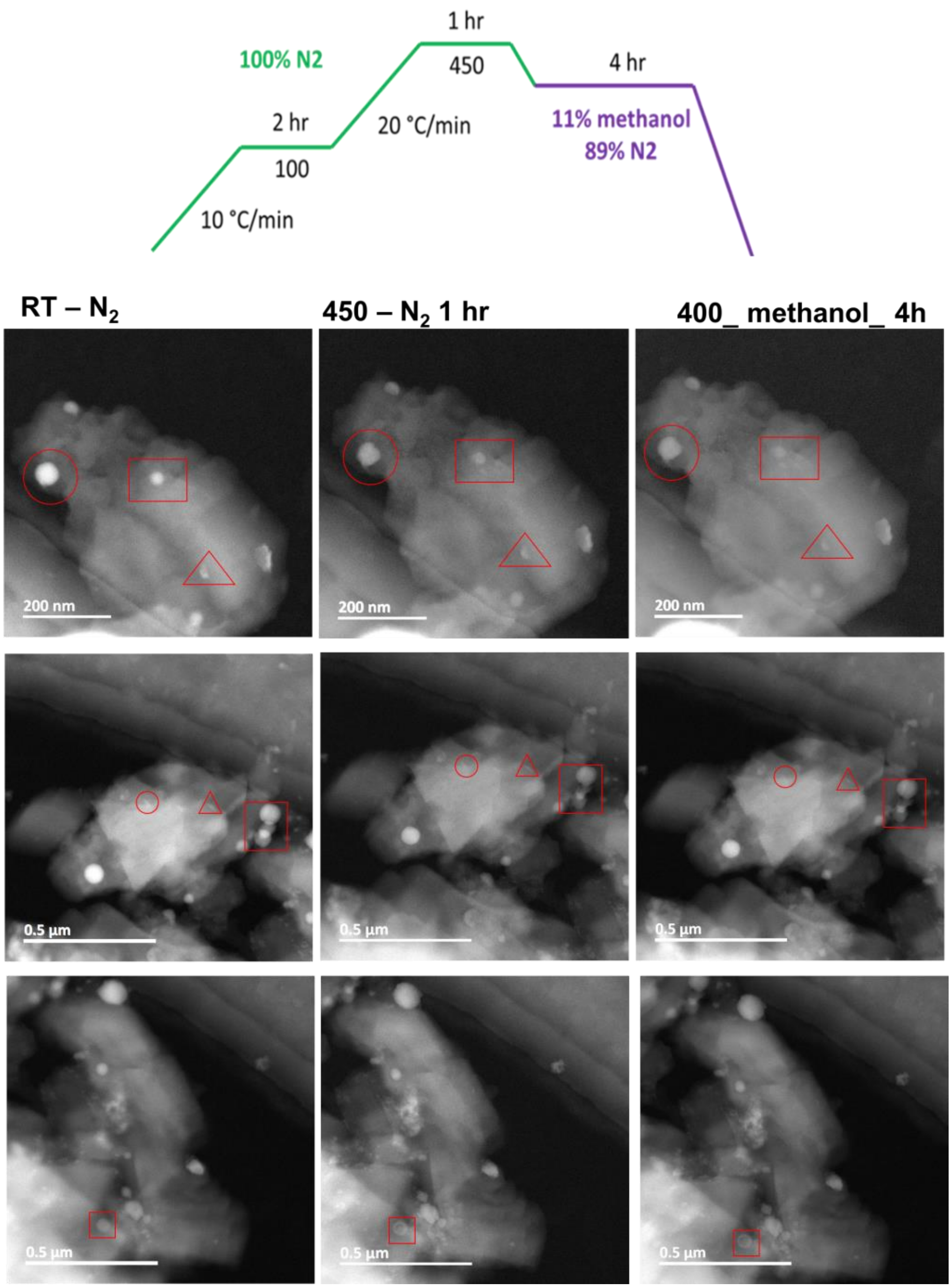

Figure S11. Evolution of the Ga/ZSM-5 sample during the in-situ STEM observation under N_2 at r.t., 450 ^oC and under methanol/N_2 mixture at 400 ^oC

The test was carried out by analyzing the different zones of the catalyst at room temperature in a flow of N_2 at room temperature, 450 ^oC and at 400 ^oC in methanol. The images show a change in the morphology of the Ga droplets due to interaction with the zeolite. This takes the form of a

gradual decrease in their intensity, sometimes resulting in their disappearance (red shapes). This phenomenon seems to depend on droplet size, as large particles do not appear to have suffered such a loss over the duration of the experiment.

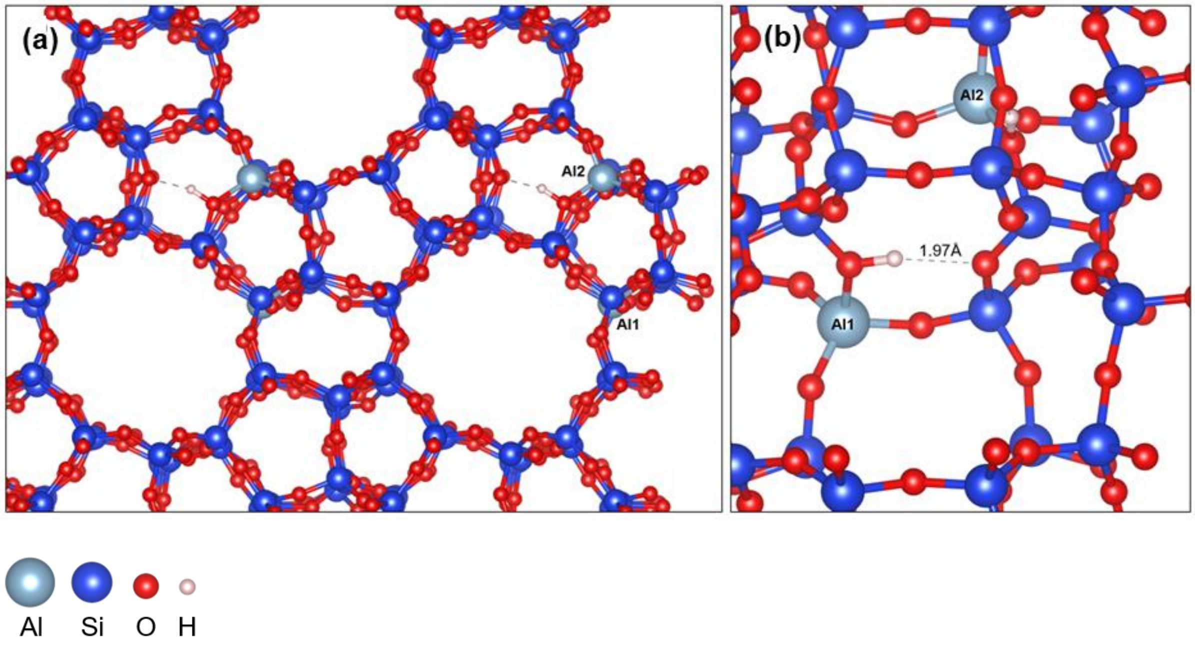

Figure S12. Periodic ZSM-5 model with 2 Al sites with corresponding protons (Si/Al=47), front (a) and side view (b). In the most stable structure, the protons form a H-bond interaction with neighboring O-atoms.

DFT calculations were performed using a periodic ZSM-5 model with a Si/Al ratio of 47 and a unit cell of 96 T atoms. The Brønsted acid sites are introduced by substituting Si by an Al atom and a charge compensating proton on a neighboring oxygen. The acid sites are in an 8-membered ring at the intersection of the straight and sinusoidal channels (**Figure S12, SI**). The distribution of the acid sites is directly affected by the synthesis procedure, but these positions have been identified as favorable positions for Al substitution in previous DFT studies due to their location in the zeolite.[1] The same periodic model was used for the Silicalite-1 but without Al sites.

The stability of small metallic Ga clusters (Ga_n) relative to bulk α-Gallium was calculated using PBE-D3(BJ) DFT electronic energies, as implemented in VASP.[2-6] Ga_1 to Ga_4 clusters in their most

stable configuration were computed (**Table S1, SI**). The stability of these clusters increases with the number of Ga atoms, converging to the sublimation energy for bulk Gallium, -290 kJ/mol$_{Ga}$. The latter DFT PBE-D3(BJ) value corresponds reasonably well with the experimental standard latent heat of sublimation, 277 kJ/mol.[7] This high value is also consistent with the high temperature required to vaporize Ga.

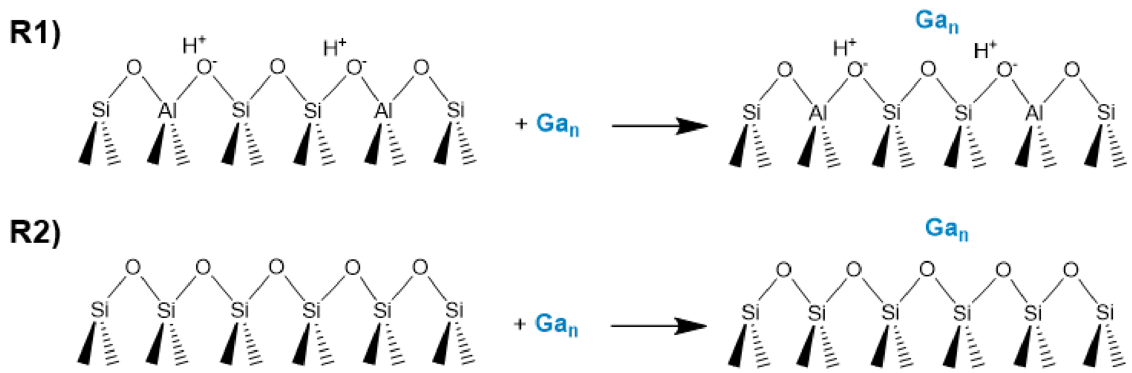

Figure S13. Reactions used to compute the adsorption energy of Ga_n cluster on ZSM-5 pores (2 Al sites) (**R1**) and on Silicalite-1 (no Al sites) (**R2**).

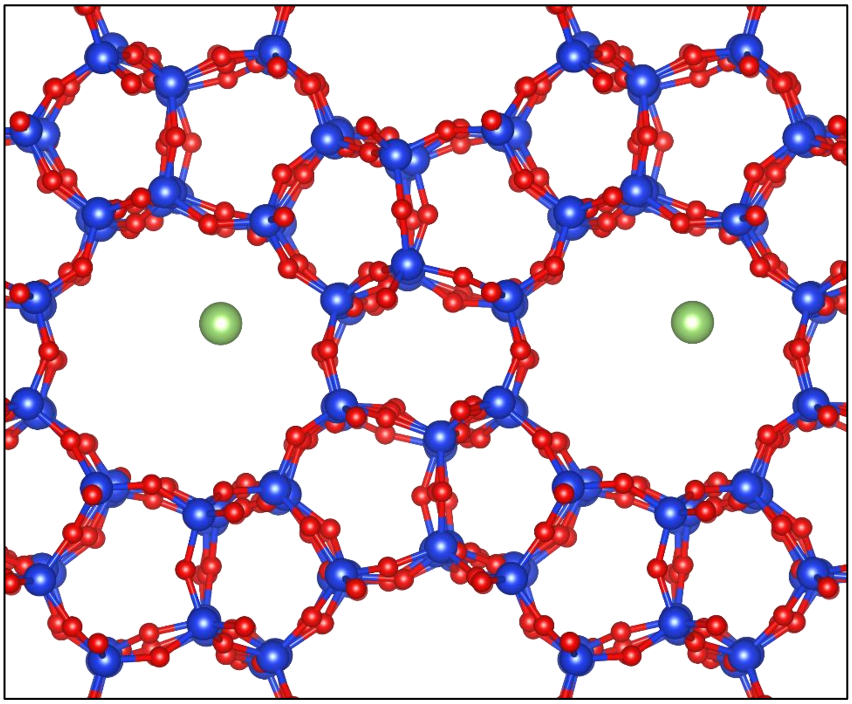

Figure S14. Structure of Ga_1/Si-1 (Si/Al=47, no Al sites).

The interaction of the small Ga clusters (Ga_1 to Ga_4) with ZSM-5 (with 2 Al sites) and with Silicalite-1 (pure Si form, with no Al sites) were computed (**Figure S13, SI** and **Table S1, SI**). These small Ga clusters interact quite strongly and specifically with the acid protons in the zeolite, with an adsorption energy of -116 $kJ/mol_{cluster}$ for Ga_1 and -143 $kJ/mol_{cluster}$ for Ga_4. The adsorption energy is much weaker in the Silicalite-1 structure, e.g., -58 $kJ/mol_{cluster}$ for Ga_1 and -105 $kJ/mol_{cluster}$ for Ga_4.

Introduction of small Ga clusters in the Silicalite-1 has a high energy penalty of 232 kJ/mol_{Ga} for Ga_1 and 111 kJ/mol_{Ga} for Ga_4 (**R2** in **Figure S13, SI**, **Figure S14, SI** and **Table S1, SI**). This energy penalty can be compensated by entropy-gain at high temperatures, $T\Delta S$, depending on the size of the cluster. The strong interaction between Ga clusters and the acid sites reduces this energy penalty for ZSM-5 to 174 kJ/mol_{Ga} for Ga_1 and 101 kJ/mol_{Ga} for Ga_4 and hence reduces the temperature for which the energy penalty is compensated by entropy gain, $T\Delta S$. More detailed calculations are required to compute this entropy gain, in particular for small Ga_2 and Ga_3 clusters.

Calculations without the D3(BJ) dispersion correction reduced the Ga_1 adsorption energy from -116 $kJ/mol_{cluster}$ to -79 $kJ/mol_{cluster}$ for Ga_1/ZSM-5 (2 Al sites) and from -58 kJ/mol to -17 kJ/mol for Silicalite-1 (no Al sites).

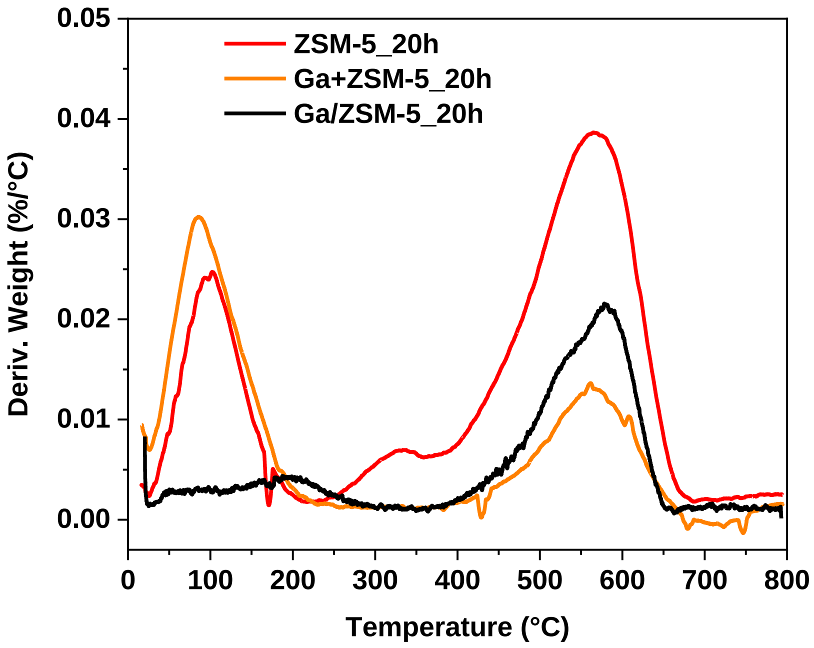

Figure S15. Weight derivatives for thermogravimetric analysis of ZSM-5, Ga/ZSM-5 and Ga+ZSM-5 after MTH on stream for 20 h.

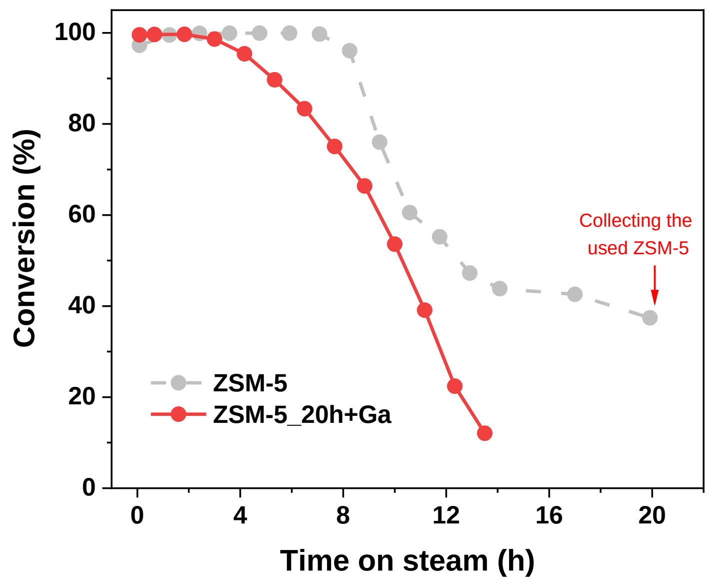

Figure S16. Catalytic data of ZSM-5_20h+Ga in methanol-to-hydrocarbon reaction. Reaction conditions: 400 °C, 50 mg of used ZSM-5 mixed with 125 mg of liquid metal of Ga, 1.4 g methanol $\cdot g^{-1}{}_{ZSM-5}\cdot hour^{-1}$.

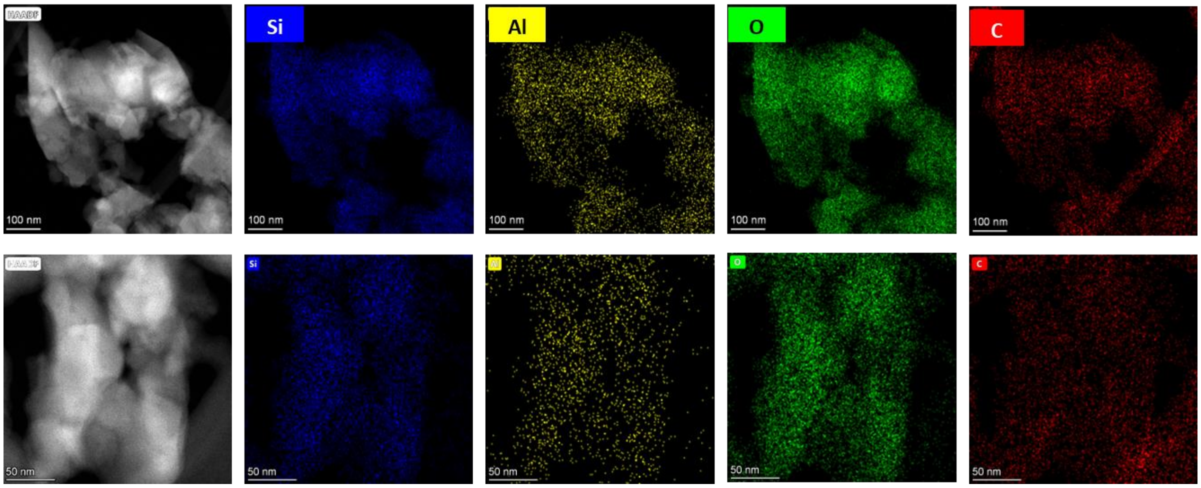

Figure S17. HRTEM and EDS elemental mapping images of H-ZSM-5 catalyst after 20 h on stream.

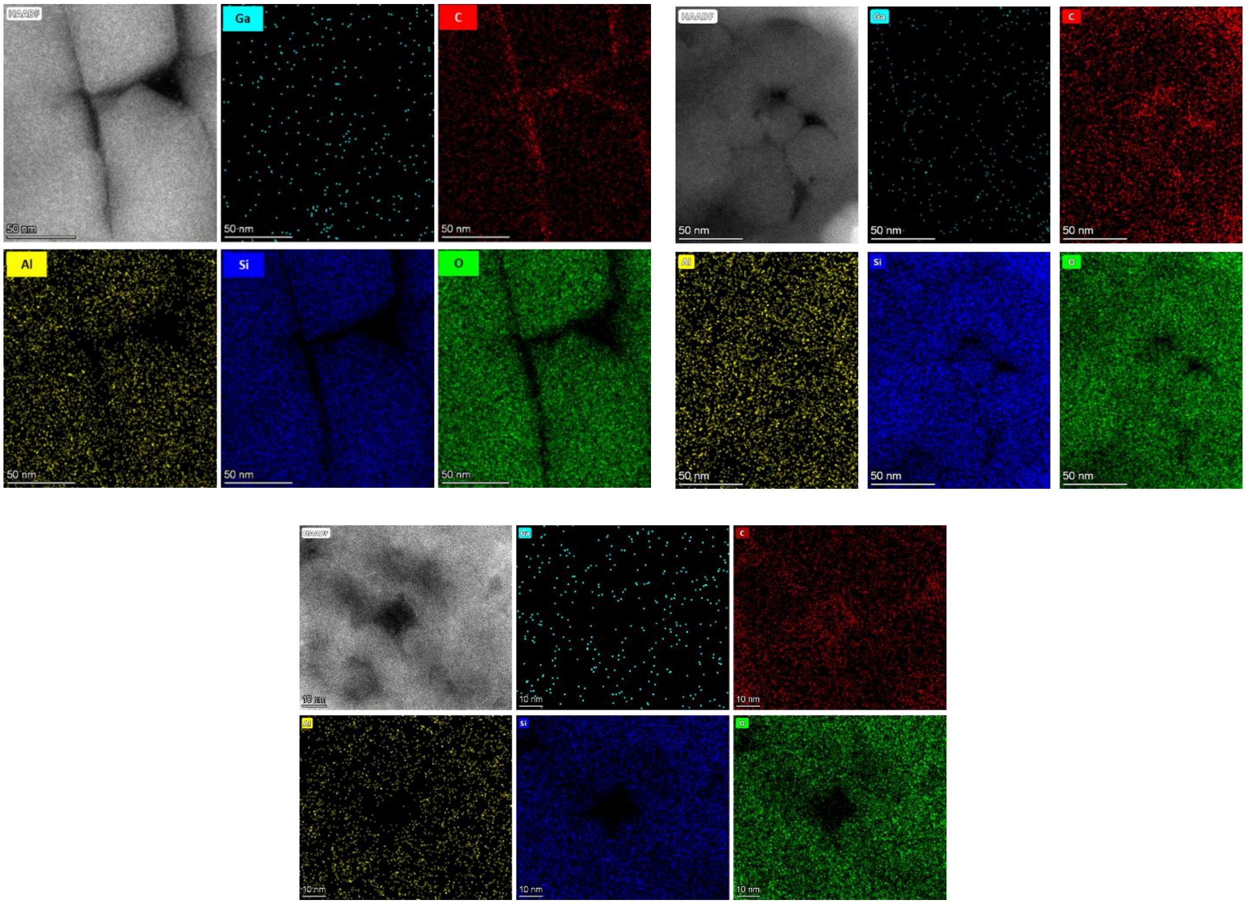

Figure S18. Representative HRTEM and EDS elemental mapping images of Ga/ZSM-5 catalyst after 20 h on stream.

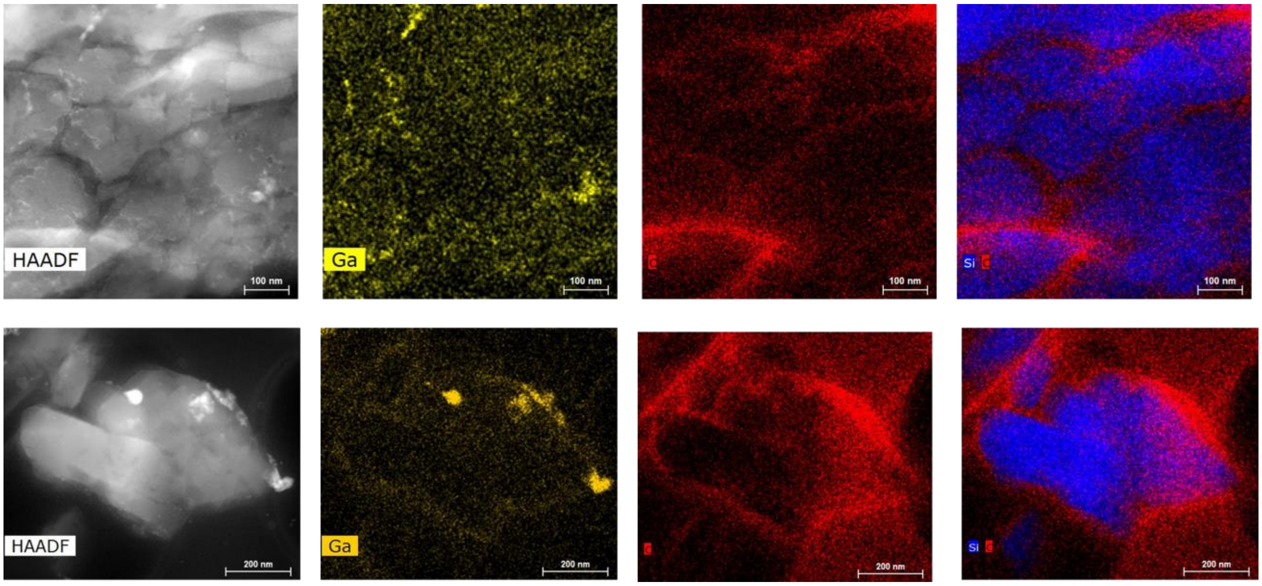

Figure S19. STEM-HAADF and EDS elemental mapping of Ga, C and superposition of Si and C in the sample ZSM-5_20h+Ga

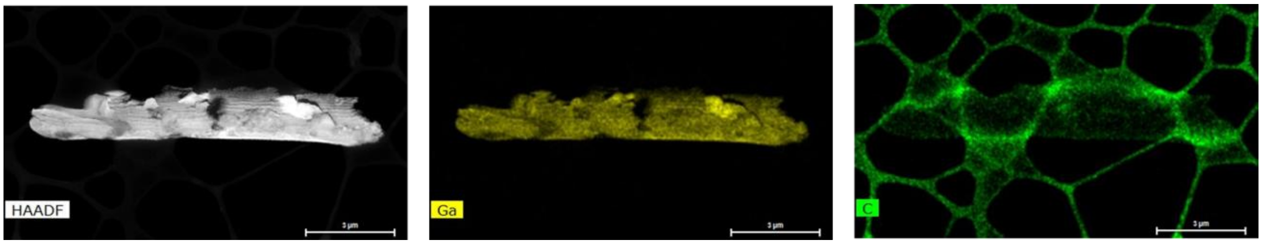

Figure S20. STEM-HAADF and EDS elemental mapping of Ga and C for Ga droplet in the sample Ga+ZSM-5_20h

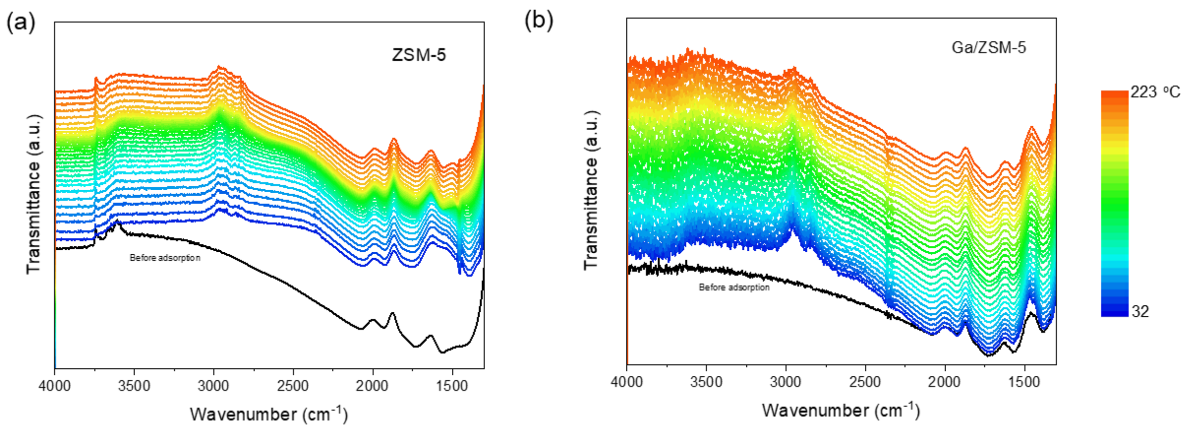

Figure S21. FTIR spectra of methanol adsorbed on ZSM-5 (a) and Ga/ZSM-5 (b) catalyst. From bottom to top the temperature goes from 32 to 223 ^{o}C.

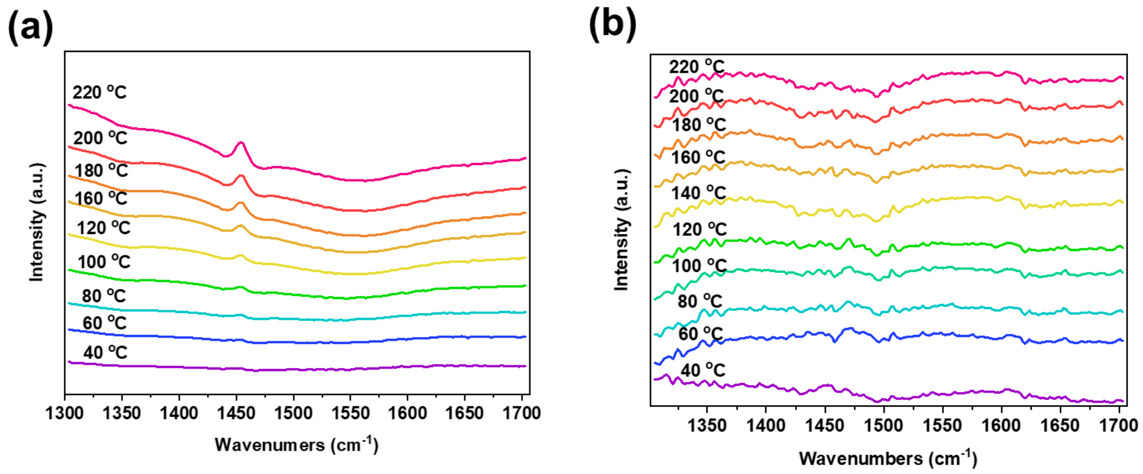

Figure S22. Evolution of the cumulative intensity of the FTIR bands during methanol conversion on ZSM-5 (a) and Ga/ZSM-5 (b).

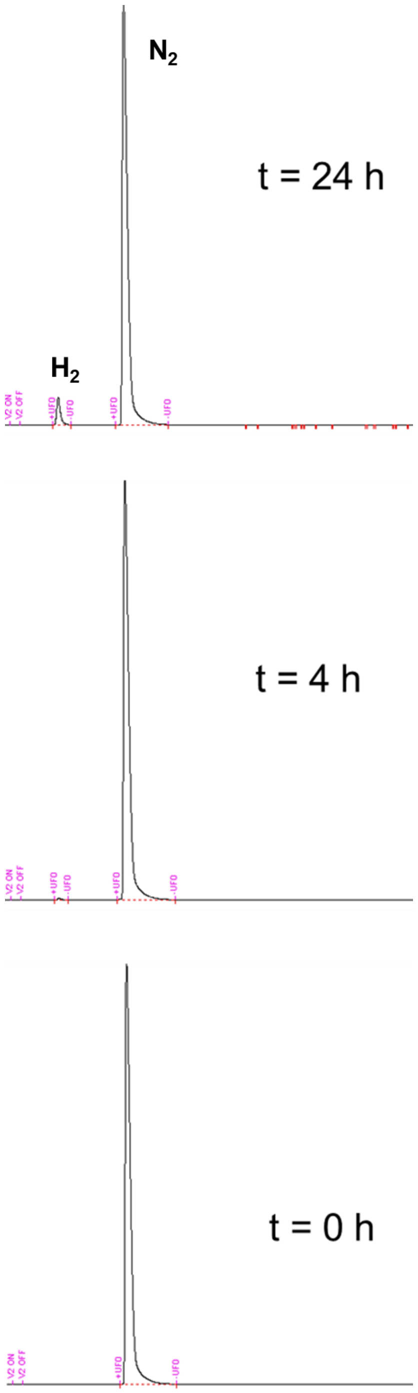

Figure S23. GC spectra of the hydrogen formation by treating ZSM-5 with Ga. 100 mg ZSM-5 mixing with 100 mg of gallium, 10 bar N_2, 250 °C.

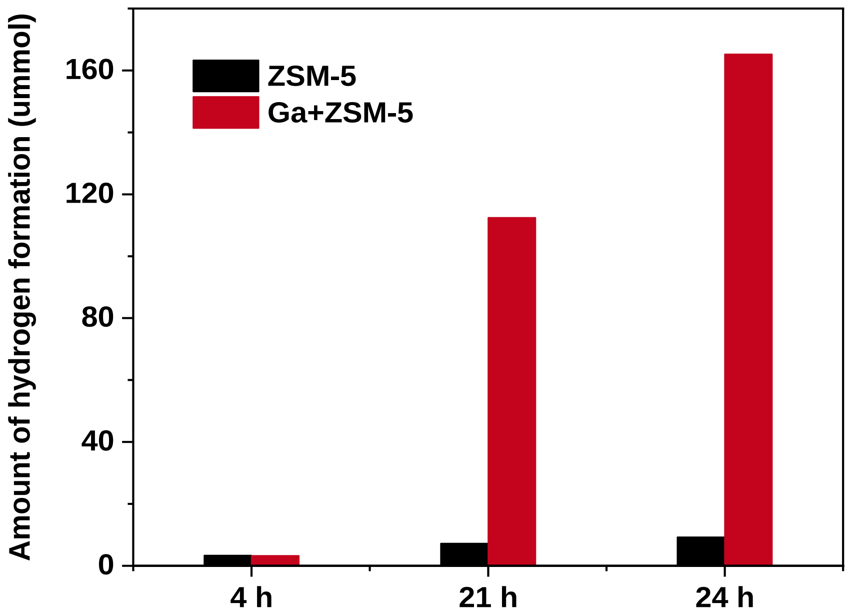

Figure S24. The amount of hydrogen produced in the presence of gallium. 100 mg of ZSM-5 or 100 mg ZSM-5 mixing with 100 mg of gallium, 10 bar N_2, 250 °C.

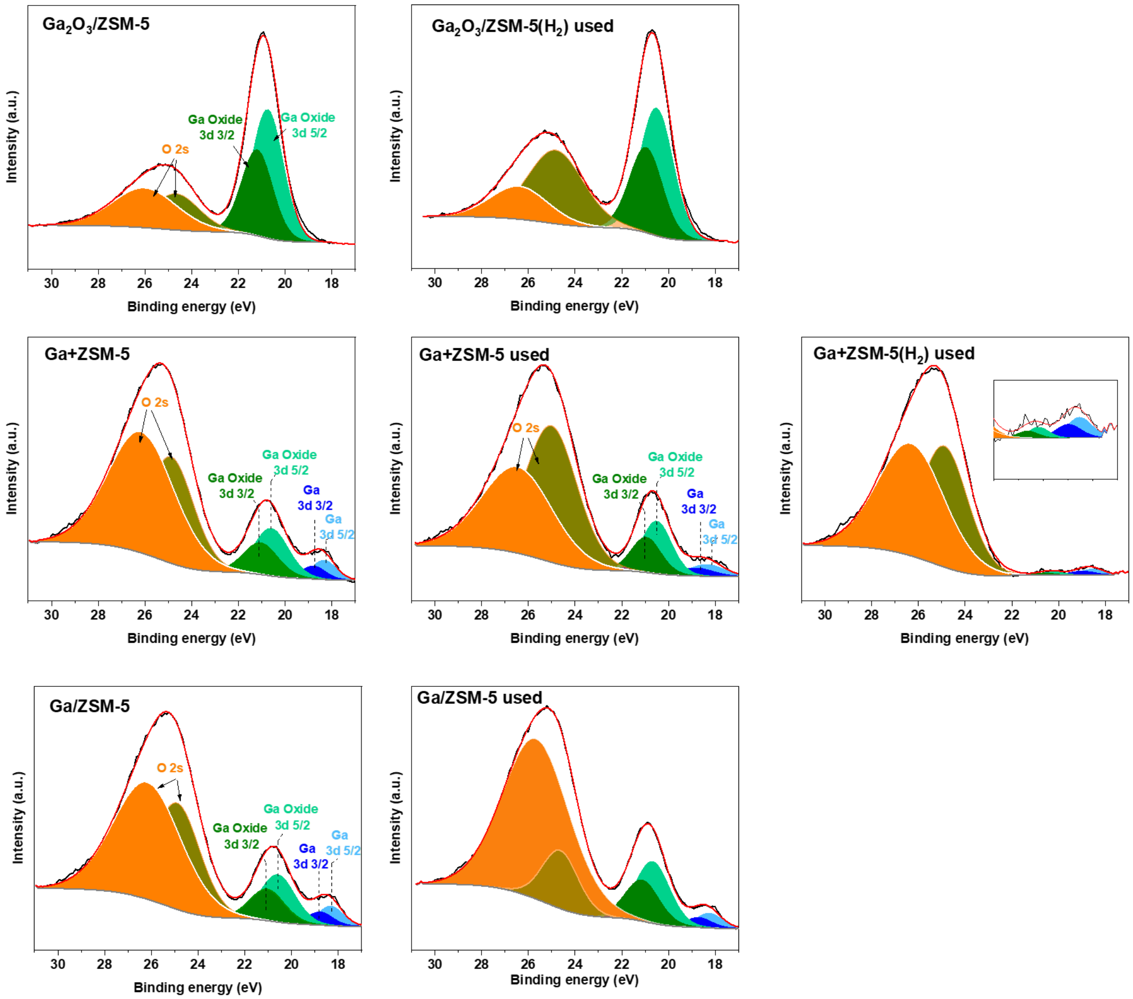

Figure S25. XPS of Ga *3d*+O *2s* of Ga_2O_3/ZSM-5, Ga+ZSM-5 and Ga/ZSM-5 before and after reaction.

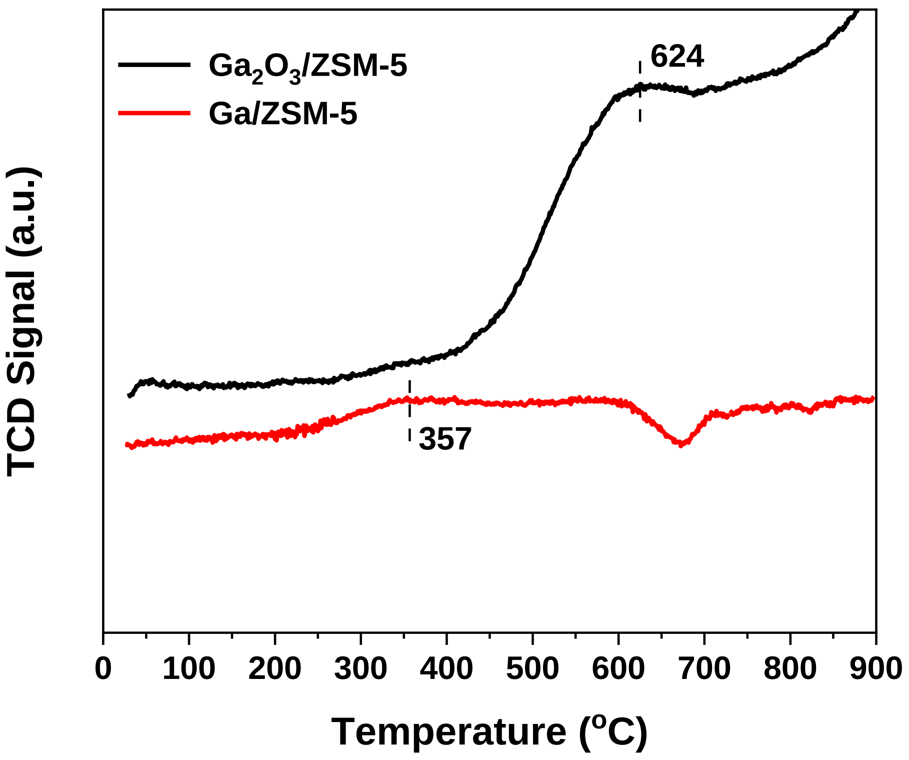

Figure S26. H_2-TPR analysis of Ga containing catalysts.

Table S1. Formation energy of gas phase Ga_n clusters, adsorption energy of Ga_n clusters on ZSM-5 (Ga_n/ZSM-5, 2 Al sites) and on Silicalite-1 (Ga_n/Si-1, no Al sites), and the formation energy of adsorbed Ga_n clusters on ZSM-5. DFT calculations performed using PBE-D3(BJ) at 0 K.

$$\text{Ga (g)} \xrightarrow{\textbf{(a)}} \text{Ga}_n$$

$$\text{Ga}_n + \text{ZSM-5} \xrightarrow{\textbf{(b)}} \text{Ga}_n/\text{ZSM-5}$$

$$\text{Ga}_n + \text{Si-1} \xrightarrow{\textbf{(c)}} \text{Ga}_n/\text{Si-1}$$

**Ga_n**	**Ga_n (g) formation energy (a) (kJ/mol_{Ga})**	**Ga_n cluster adsorption energy on ZSM-5 (b) ($kJ/mol_{cluster}$)**	**Ga_n cluster adsorption energy on Si-1 (c) ($kJ/mol_{cluster}$)**	**Ga_n/ZSM-5 formation energy (a)+(b/n) (kJ/mol_{Ga})**
**Ga_1**	0	-116	-58	-116
**Ga_2**	-82	-127	-92	-146
**Ga_3**	-124	-142	-98	-171
**Ga_4**	-153	-143	-105	-189
**Ga_{Bulk}**	-290*			

*$\Delta H_{sublimation}$ (Ga) = 277 kJ/mol_{Ga} (experimental value at 1 atm).

Table S2. Acidic properties of activated and used ZSM-5 and Ga/ZSM-5 determined by FTIR spectroscopy of adsorbed pyridine.

	150 ºC			350 ºC		
	Bronsted mmol/g	Lewis mmol/g	C_L/C_B	Bronsted mmol/g	Lewis mmol/g	C_L/C_B
ZSM-5	0.82	0.22	0.27	0.58	0.18	0.31
ZSM-5_20h	0.23	0.07	0.30	0.14	0.03	0.21
Ga/ZSM-5	0.48	0.23	0.48	0.33	0.16	0.48
Ga/ZSM-5_20h	0.30	0.21	0.70	0.17	0.14	0.82
